# “Cueing” for Levodopa-Induced Dyskinesias in Parkinson’s Disease

**DOI:** 10.3389/fneur.2016.00237

**Published:** 2016-12-23

**Authors:** Eva Schaeffer, Gerd Linke, Daniela Berg

**Affiliations:** ^1^Department of Neurology, Christians-Albrechts-University of Kiel, Kiel, Germany; ^2^ZAR, Department for Physical Therapy, University of Tuebingen, Tuebingen, Germany; ^3^Department of Neurodegeneration, Hertie Institute of Clinical Brain Research, University of Tuebingen, Tuebingen, Germany

**Keywords:** Parkinson’s disease, cueing, dyskinesia, training, silly walks

## Abstract

We describe the case of an early-onset Parkinson’s disease (PD) patient, suffering from a severe and unusual dyskinetic gait pattern, earlier described as “Silly walk.” On presentation, the 58-year-old patient showed a painful, bizarre dyskinetic gait disorder, resulting in a significantly impairment of her social life. We developed an individual conservative training method for the patient, using “Cueing mechanisms,” well known for treatment of Freezing in PD, to overcome her dyskinetic gait pattern. An impressive improvement was seen after the use of visual and acousting cues. We, therefore, conclude that it might be important for PD patients to recognize these specific movement abnormalities and start early with individualized training methods. Moreover, especially for “Silly walk” individuals, “Cueing” strategies can be an important new basis of conservative training methods for dyskinesia.

## Background

Levodopa-induced dyskinesia is one of the most bothersome side-effects of dopaminergic treatment in patients with Parkinson’s disease (PD). Treatment strategies include mainly optimization of medical treatment and deep brain stimulation, whereas experiences with specific exercise strategies are still missing. Contrary, for other motor symptoms in PD, specific conservative training methods are frequently and successfully used in daily clinical routine. In particular, the use of specific external visual or acoustic stimuli, so called “Cueing,” has been implemented as an important basis for the treatment of gait freezing in Parkinson’s disease.

It has been shown that dyskinesias occur more often in early-onset PD patients (1), especially in those with a specific underlying genetic pathology (2). In 2011, Ruzicka et al. described four cases of dyskinesias in early-onset PD patients, who was presented with a very unusual dyskinetic gait pattern, which they described as “Silly walks” (3). We here present the case of a young women with early-onset PD, who suffered from a similar severe dyskinetic gait disturbance and showed an impressive improvement while exercising with specific “Cueing” strategies.

## Introduction

Our patient developed symptoms of PD at age 38, starting with slowing of handwriting on the right side. A mutation in the Parkin gene was verified. Dopaminagonist treatment was started after about 1 year and right from the beginning a therapy with Levodopa was added. The patient first recognized changes in her gait pattern after about 13 years of levodopa therapy. In the following years, she developed a severe dyskinetic gait disturbance in the right leg, with a monomorph flexion in the hip and a kicking extension in the knee while walking. This gait disturbance presented mainly as on-dyskinesia, sometimes as biphasic dyskinesia, most of the time lasting more than 50 percent of the daytime. When we first met the patient (20 years after PD onset), she reported pain in her right hip, with the dyskinetic kick and reflexion of her leg being sometimes so extensive that she was worried about her hip joint. Moreover, the bizarre presentation of her gait resulted in a considerable impairment of social life.

## Treatment

The impression that the gait pattern of our patient was extremely monomorphic lead us to the hypothesis that Cueing mechanisms might change this pattern. The training was started about 60 min after the last levodopa intake of the patient, when the dyskinetic gait pattern was strongly pronounced.

We started with a visual cueing method, using colored foot prints with very small distances, to prevent large dyskinetic steps. Starting from the first trial, the patient was able to clearly reduce the dysinetic leg movements by stepping on the colored pathway given. After about 20 min of training, the footsteps were removed and the patient was asked to imagine the colored footsteps while walking. As demonstrated in the video, the patient was able to control her gait pattern, but only at that place in the hall, where the colored footsteps were placed before. The visual cueing training was continued for 40 min, with a stable and reproducible response in every training trial.

In a second approach, we tried to find an acoustic cueing effect, hypothesizing that a very fast melodic rhythm might induce very short footsteps and, therefore, prevent again the large dyskinetic steps. Interestingly, after the music started, the patient always needed a few seconds to “find the rhythm,” followed by an impressive sudden change of her gait to a nearly normal pattern. The acoustic cueing was repeated for 30 min and once again, the patient could change her gait pattern in every trial.

Following the first training, the patient was asked to exercise with the new cueing strategies for a period of 6 weeks at home, three times per week for 45 min, supported by a physiotherapist and video monitoring. Just like in the first training setting at our clinic application of the cueing strategies always immediately changed the dyskinetic movements in the training environment.

## Discussion

Although the “funny” gait disturbances in dyskinetic patients are frequently observed and described by clinicians, specifically targeted therapies, in particular conservative training methods, have not been developed so far. However, this movement disturbance frequently has a very important influence on social life, especially in the case of monomorphic awkward movements, which are often misinterpreted as psychogenic disorders. Moreover, these dyskinesias can increase the risk of secondary complications, such as falls or arthrosis.

“Silly walks” differ from other dyskinetic movements disorders as they have a stereotypic character, which makes them ideal for the targeted use of cueing strategies. In our patient marked improvement leading to an almost completely normal gait pattern was observed. However, it has to be taken into account that although the observed effect was impressive, it can not be generalized yet. Longitudinal studies with a larger cohort are needed to validate the feasibility and observe the long-term effect of this method for PD patients. Moreover, our patient reported difficulties in transferring the cueing strategies into daily life activities. While the described cueing mechanisms alleviated always successfully her dyskinesia in specific environments, for example at home or in the gym, it was difficult for her to implement these strategies in her daily life. This may be due to the fact that she has been living with this form of dyskinesia for more than 7 years, before the cueing strategy was started. It therefore seems essential to recognize these gait patterns early, especially in young-onset PD patients, and immediately start with a targeted training to prevent fixation of this movement disturbance.

## Concluding Remarks

This case highlights the importance of recognizing individual movement abnormalities in PD, especially in early-onset PD patients, which might suffer from unusual and extremely bothersome dyskinetic gait patterns as described above. Early detection of these movement disorder might open up the ability for early conservative training methods and, in particular, the use of cueing mechanisms, which has not been implemented in the therapy of dyskinesia so far.

## Ethics Statement

Case report, no ethical approval procedure needed. The patient gave her written informed consent for the publication of the manuscript and video.

## Author Contributions

ES and DB were involved in the design and performance of the training and acquisition of information, writing of the first draft, and revising of the work. GL contributed to the design and performance of the training and acquisition of information as well as revising of the work. All authors contributed to and have approved the final manuscript.

## Conflict of Interest Statement

ES and GL have nothing to declare. We did not at any time receive payment or services from a third party for any aspect of the submitted work. DB reports grants from Janssen Pharmaceutica, grants from Michael J. Fox Foundation, grants from German Parkinson’s Disease Association (dPV), grants from BMWi, grants from BMBF, grants from Parkinson Fonds Deutschland GmbH, grants and speaker’s honoraria from and consultancies for UCB Pharma GmbH, grants and speaker’s honoraria from TEVA Pharma GmbH, grants from and consultancies for Novartis Pharma GmbH, grants from Boehringer Ingelheim Pharma GmbH, grants and speaker’s honoraria from and consultancies for Lundbeck; outside the submitted work.

## Supplementary Material

Video S1 **Segment 1 – First presentation**. Our patient presented with a dyskinetic gait pattern, occuring mainly as peak-dose dyskinesia and sometimes as biphasic dyskinesia. The video is filmed about 40 min after levodopa intake. **Segment 2 – Visual “cues.”** The training was started about 60 min after the last levodopa intake, when the patient showed the maximum expression of her dyskinetic gait pattern. The patient was asked to follow colored footprints while walking and her dyskinetic movements where immediately reduced. **Segment 3 – Imagination of visual “cues.”** The foot prints were removed and the patient was asked to imagine them on the floor. She was still able to control the dyskinetic movements, but only at that part of the hall where the footprints were placed before. **Segment 4 – Acoustic “cues.”** A few seconds after the music started the patient showed a sudden improvement of her dyskinetic leg movements.

## References

[1] Kostic V Przedborski S Flaster E Sternic N Early development of levodopa-induced dyskinesias and response fluctuations in young-onset Parkinson’s disease. Neurology (1991) 41(2 Pt 1):202–5. 10.1212/WNL.41.2_Part_1.202 1992362

[2] Klein C Pramstaller PP Kis B Page CC Kann M Leung J Parkin deletions in a family with adult-onset, tremor-dominant parkinsonism: expanding the phenotype. Ann Neurol (2000) 48(1):65–71. 10.1002/1531-8249(200007)48:1<65::AID-ANA10>3.0.CO;2-L 10894217

[3] Ruzicka E Zárubová K Nutt JG Bloem BR “Silly walks” in Parkinson’s disease: unusual presentation of dopaminergic-induced dyskinesias. Mov Disord (2011) 26(9):1782–4. 10.1002/mds.23667 21495073 PMC3139772

